# Synthesis of 2*RS*,4*RS*-1-[2-Phenyl-4-[2-(2-trifluromethoxy-phenoxy)-ethyl]-1,3-dioxolan-2-yl-methyl]-1*H*-1,2,4-triazole Derivatives as Potent Inhibitors of Brassinosteroid Biosynthesis

**DOI:** 10.3390/molecules17044460

**Published:** 2012-04-13

**Authors:** Kazuhiro Yamada, Yuko Yoshizawa, Keimei Oh

**Affiliations:** Department of Biotechnology, Faculty of Bioresource Sciences, Akita Prefectural University, 241-438, Shimoshinjo Nakano, Akita 010-0195, Japan

**Keywords:** brassinosteroid, brassinosteroid biosynthesis inhibitor, plant growth regulators

## Abstract

Brassinosteroids are important phytohormones that affect many aspects of plant growth and development. In order to manipulate brassinosteroid levels in plant tissues by using specific biosynthesis inhibitors, we have carried out a systemic search for specific inhibitors of brassinosteroid biosynthesis. Synthesis of triazole derivatives based on the ketoconazole scaffold revealed a series of novel brassinosteroid biosynthesis inhibitors (the **YCZ** series). To explore the structure-activity relationships of this synthetic series, we now report the synthesis of new triazole derivatives with different aromatic structures at position 2 of 1,3-dioxolane skeleton. We found that the variation of aromatic substituent significantly affect the inhibitory potency. Structure-activity relationships studies indicated that 4-chlorophenyl analogue is the most potent inhibitor of BR biosynthesis with an IC_50_ value approximately 0.12 ± 0.04 µM, while a bulky biphenyl group exhibited a great negative effect on promoting the inhibitory potency with an IC_50_ larger than 10 µM.

## 1. Introduction

Brassinosteroids (BRs), a class of polyhydroxysteroids widely distributed in plant kingdom, are potent phytohormones that affect many aspects of plant growth and development [1]. Physiological studies have demonstrated that BR can induce diverse cellular responses such as stem elongation, pollen tube growth, leaf bending, root inhibition, induction of ethylene biosynthesis and fruit ripening, and stress tolerance [1,2,3]. The identification of BR biosynthetic mutants of *Arabidopsis*, tomato, rice and pea established that BR is a new class of phytohormone with an essential role in plant growth and development [4,5,6,7]. Since then, efforts have been made to control the BR biosynthesis in plant tissues by genetic approaches and available evidence indicates that mutations in BR biosynthesis may be a means to improve biomass production [8,9]. Consequently, development novel methodology to manipulate BR levels in plant tissues attracts great research interests.

An alternative method to control the BR levels in plant tissues is the use of specific inhibitors that target the enzymes responsible for BR biosynthesis. BR biosynthesis inhibitors have consequently become highly viable candidates for plant growth regulators. Asami and Yoshida reported the discovery of brassinazole, the first synthetic BRs biosynthesis inhibitor (Brz series) [10]. Subsequent studies on the mode of actions of Brz provided important information about the functions of BRs [11,12]. Recent progress on molecular functional analysis enzymes of BR biosynthesis provided insight evidences that P450 enzymes play key roles in the process of hydroxylation of BR [13,14,15]. Accordingly, strategies for designing P450 inhibitors can be applied to the identification of BR synthesis inhibitors. Cytochrome P450 inhibition mechanisms have been studied in considerable detail [16], triazole derivatives have been demonstrated widespread utility as inhibitors of P450s, due to the intrinsic affinity of the nitrogen electron pair in heterocyclic molecules for the prosthetic heme iron [17].

**Figure 1:**
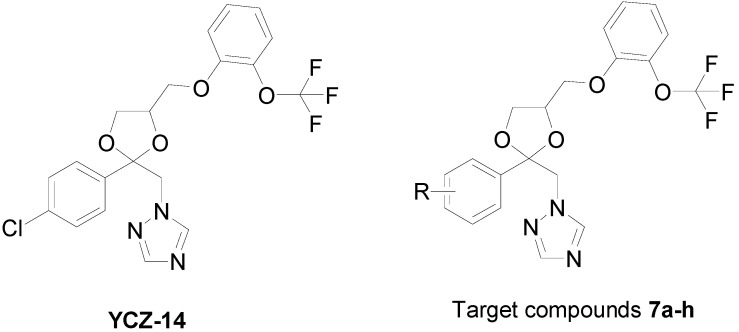
Chemical structure of **YCZ-14** and general structure of the target compounds **7a**–**h**.

Our research interests are in developing novel plant hormone biosynthesis inhibitors and the use of these compounds to explore the functions of plant hormones in plant growth and development [18,19,20,21]. In the previous work, we have shown that 2*RS*,4*RS*-1-[2-(4-chlorophenyl)-4-[2-(2-trifluoromethoxy-phenoxy)-ethyl]-1,3-dioxolan-2-yl-methyl]-1*H*-1,2,4-triazole (**YCZ-14**, Figure 1) is a potent inhibitor of BR biosynthesis [22]. 

The biological activity of this synthetic series (**YCZ** series) was evaluated by testing the ability of the compounds on retardation stem elongation of *Arabidopsis* seedlings. The compound-induced hypocotyls dwarfism were counteracted by the co-application of 10 nM brassinolide, the most potent BR, but not 1 μM GA_3_, which produced seedlings that resembled BR-deficient mutants. In order to further study the structure-activity relationships of **YCZ** series, we report herein the synthesis of new triazole derivatives with different aromatic structure at the position 2 of the 1,3-dioxolane moiety to mimic the partial structure of 4-chlorophenyl moiety found in **YCZ-14** (The general structure of target compounds **7a**–**h** is shown in Figure 1). Structure-activity relationships of newly synthesized compounds were discussed.

## 2. Results and Discussion

### 2.1. Chemistry

**Scheme 1:**
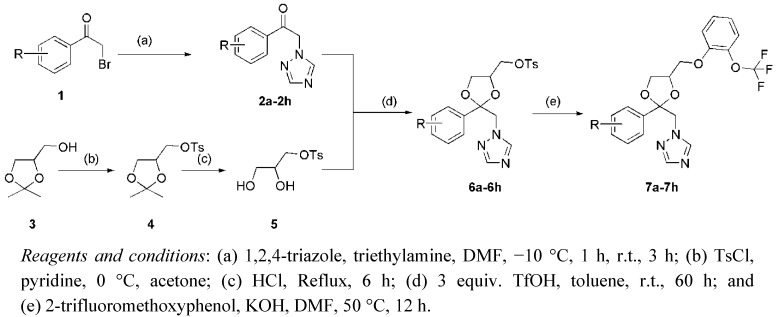
The chemical synthesis of target compounds.

Target compounds **7a**–**h** were prepared according to a synthetic route (Scheme 1) as we previously described [22]. 

The key transformation of **2a**–**h** with compound **5** consisted of four steps: (1) formation of ethanones **2a**–**h**; (2) tosylation of isopropylideneglycerol **3**; (3) deprotection of isopropylidene ketal **4**; and (4) ketal formation to generate **6a**–**h**. Compounds **2a**–**h** were prepared by reacting different kinds of commercially available *a*-bromoketones **1** with triazole in DMF using a method that we described previously [23]. The alkylation of isopropylidene glycerol **3** was achieved using a method we described previously [22], and hydrolysis with 1 M HCl in MeOH yielded glyceryl tosylate **5**. Ketal formation to generate **6a**–**h** were carried out using three equivalents of trifluoromethanesulfonic acid (TfOH) in toluene at room temperature for 60 h, according to a method previously described [22]. All of the compounds synthesized in this work consist of four stereoisomers, and they were subjected to biological studies without further purification.

### 2.2. Bioassay Methods

The bioassay used for determination the activity of BR biosynthesis inhibitors was carried out by a method as we described previously [22]. *Arabidopsis* BR synthesis-deficient mutants such as *dwarf 1 *show remarkable dwarfism and the opening of the apical hook of cotyledons in the dark [4]. This unique de-etiolation in the dark phenotype has been used for screening for BR biosynthesis inhibitors [24]. In the present study, we adapted this assay method to determine the effects of test compounds on hypocotyls elongation of *Arabidopsis* seedlings grown in the dark, and we co-applied BL and GA with the test compounds to determine the reversibility of their effects. With this assay system, we evaluated the biological activities of synthesized compounds.

### 2.3. Biological Activities of Newly Synthesized Brassinosteroid Biosynthesis Inhibitors

The chemical structures of compounds applied for biological studies are shown in Table 1. To identify the aromatic chemical structure at position 2 of 1,3-dioxolane ring responsible for the retardation of *Arabidopsis *stem elongation*, *various aromatic substituent were introduced to the inhibitors **7a**–**h**. 

**Table 1:** Inhibitory activity of triazole derivatives on *Arabidopsis* seedling growth. 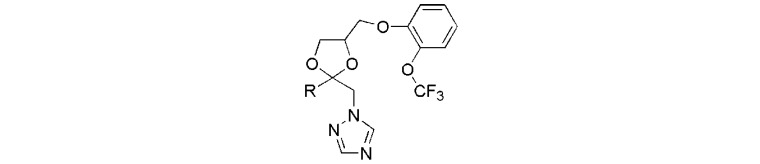

No.	-R	Inhibition of *Arabidopsis*stem elongation (IC_50_, μM) ^a^
**7a**	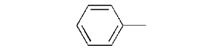	0.46 ± 0.04
**7b**	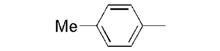	0.26 ± 0.05
**7c**	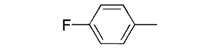	0.21 ± 0.01
**7d**	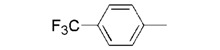	0.73 ± 0.06
**7e**	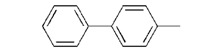	>10
**7f **	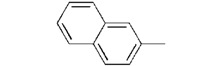	2.63 ± 0.39
**7g **	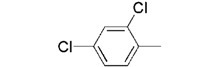	0.19 ± 0.05
**7h **	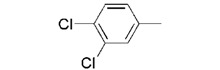	2.40 ± 0.22
**YCZ-14**	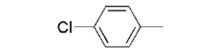	0.12 ± 0.04
**Brz**	‒	0.73 ± 0.13

^a^ The IC_50_ values of the test compounds for the inhibition of *Arabidopsis* stem elongation were calculated as described in experiment section. All of the experiments were performed at least in duplicate to establish the repeatability.

We used **YCZ-14** and **Brz** as positive controls. A phenyl analogue (compound **7a**) was used as a baseline reference for structure-activity relationships discussions. The concentrations of all of the test compounds as well as **Brz** were assigned to be 0, 0.01, 0.05, 0.1, 0.5, 1 and 10 μM, and the IC_50_ values were calculated accordingly. As shown in Table 1, compound **7a** exhibits inhibitory activity on retarding hypocotyls elongation of *Arabidopsis* seedling grown in the dark, with an IC_50_ value of 0.46 ± 0.04 μM, while the IC_50_ of **YCZ-14 **was 0.12 ± 0.04 and **Brz** was 0.73 ± 0.13 μM, respectively. This result indicates that the inhibitory potency of **YCZ-14** (4-chlorophenyl analogue) is stronger than that of **7a** and that a mono substituent at position 4 of the phenyl moiety may promote the inhibitory activity. To verify this possibility, we further introduce 4-methylphenyl, 4-fluorophenyl and 4-trifluoromethylphenyl moieties into the inhibitor (analogues **7b**–**d**) to evaluate their effect on inhibitory activity. 

We found that analogues with methyl and fluorine atom substituents at position 4 of the phenyl ring (compounds **7b**,**c**) have a positive effect on promoting the inhibitory activity compared with that of **7a**, with IC_50_ values of 0.26 ± 0.05 and 0.21 ± 0.01 μM, respectively. Interestingly, introducing a 4-trifluoromethylphenyl moiety (compound **7d) **to the position 2 of 1,3-dioxolane, however, showed a significant negative effect on promoting inhibitory activity, with an IC_50_ value approximately 0.73 ± 0.06 μM. It is worthwhile to note that compound **7d** shares the common 4-trifluoromethylphenyl moiety with Brz220, the most potent inhibitor of BR biosynthesis inhibitor reported by Asami*et al.* (the **Brz** series) [25]. Data obtained in this work suggests that the structure requirements for these two synthetic series (**Brz** and **YCZ**) on inhibition of BR biosynthesis are different. This observation implies the binding site of **YCZ** series may be different from **Brz** series. In order to further determine the structure-activity relationships of **YCZ** synthetic series, we next introduced a bulky aromatic moiety to the inhibitor. As shown in Table 1, the IC_50_ value of the biphenyl analogue **7e** and naphthalene analogue **7f** are greater than 10 and 2.63 ± 0.39 μM, respectively. This result indicates that a bulky aromatic moiety significantly reduces the inhibitory activity. Although the variation of aromatic structure greatly affects the inhibitory activity of this synthetic series, the 4-chlorophenyl analogue (**YCZ-14**) is the most potent inhibitor. This observation suggests that the substituent(s) of chlorine atom on the phenyl moiety enhance the inhibitory activity of this synthetic series. Thus, we next synthesized 2,4-dichlorophenyl analogue **7g** and 3,4-dichloro-phenyl analog **7h** for further structure-activity relationships studies. We found the IC_50_ values for **7g** and **7h** are 0.19 ± 0.05 and 2.40 ± 0.22 μM, respectively. Although the structural difference of these two analogues is the position of the chlorine atom, the inhibition potencies of these two analogues are quite different. This result indicates that the position of the chlorine atom on the phenyl ring is sensitive to the binding site. Compared the inhibitory potency of analogues reported in this work with **Brz**, we found the potency of several **YCZ** analogues are greater than **Brz**, except **7d**, **7e**, **7f** and **7g**. Nevertheless, among the compounds studied in this work, **YCZ-14** is the most potent inhibitor on retardation stem elongation of *Arabidopsis *seedlings.

It is known that GA biosynthesis inhibitors, such as paclobutrazol, retard the stem elongation of many plant species by blocking *ent*-kaurene oxidation and also mildly affect other cytochrome P450 mono-oxygenases [25]. This retardation can be rescued by the application of GA. In order to rule out the GA biosynthesis inhibitor among these analogues, we tested the effects of brassinolide, the most biologically active BR, and GA on the recovery of chemical induced dwarfism of *Arabidopsis* seedlings grown in the dark. Accordingly, we selected analogues with IC_50_ values less than 1 μM for studying the mode of actions. As shown in Table 2, compounds **7a** to **7d** and **7g** were subjected to the bioassay at a concentration of 0.5 μM and *Arabidopsis* seedlings were grown in the presence of BL (10 nM) or GA (1 μM) for 5 days in the dark. Data were expressed in percentage by the comparison of none chemical treated control.

**Table 2:** Retardation of *Arabidopsis* seedling growth by triazole derivatives and rescue of growth by BL and GA. 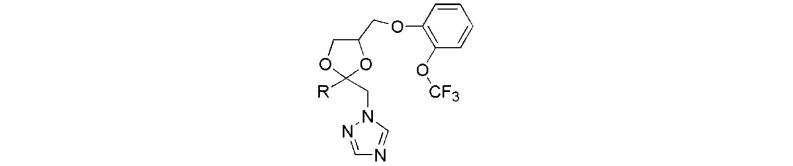

No.	-R	Hypocotyl length, % relative to untreated *Arabidopsis* seedlings (%)		
		Chem *.	Chem. + BL (10 nM)	Chem. + GA (1 µM)
**Control**	-	100	114.3 ± 8.0	104.3 ± 5.4
**7a**	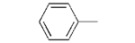	45.9 ± 2.5	99.8 ± 4.4	48.0 ± 3.7
**7b**	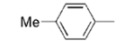	37.6 ± 3.2	95.4 ± 4.9	43.8 ± 3.7
**7c**	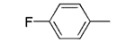	32.5 ± 3.2	101.2 ± 4.5	39.7 ± 2.5
**7d**	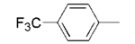	56.7 ± 1.1	103.3 ± 4.5	51.8 ± 2.8
**7g**	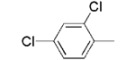	21.2 ± 2.3	104.1 ± 3.3	31.5 ± 3.7
**YCZ-14**	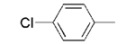	18.2 ± 2.0	97.0 ± 3.9	25.5 ± 3.0
**Brz**	‒	56.0 ± 3.3	63.0 ± 6.1	61.8 ± 2.3

* Data obtained from 20 seedlings grown in the dark. Chem. was assigned to be at a final concentration of 0.5 µM. All the experiments were done at least three times to establish the repeatability.

**Figure 2:**
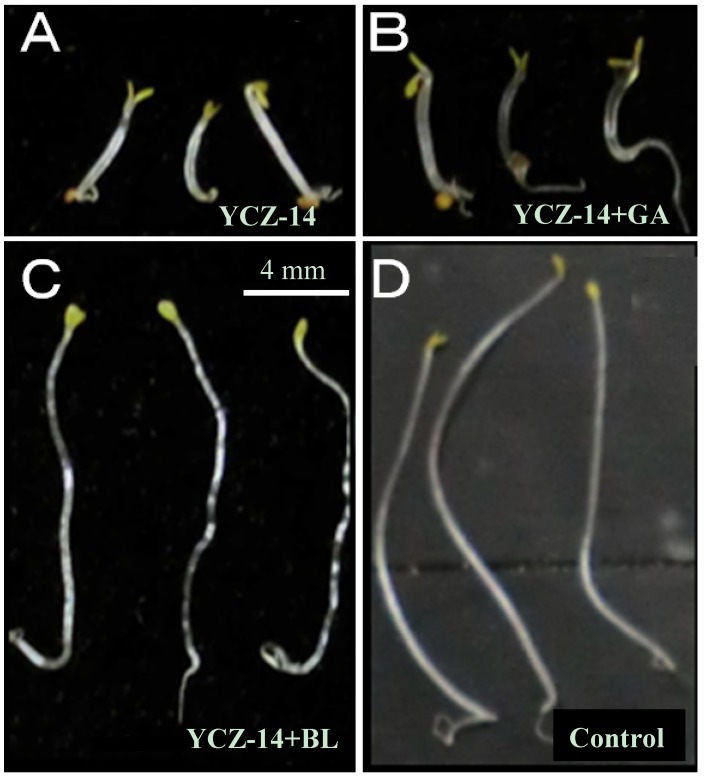
Effect of **YCZ-14** on 5 days old *Arabidopsis* seedlings grown in the dark. A: **YCZ-14** (0.5 µM); B: **YCZ-14** (0.5 µM) + GA (1 µM); C: **YCZ-14** (0.5 µM) + BL (10 nM); D: Control.

As shown by the data, in the presence of BL (10 nM) or GA (1 μM), the average hypocotyls length of *Arabidopsis* seedlings were approximately 114.3 ± 8.0 and 104.3 ± 5.4%, respectively. This result indicates that BL and GA stimulate hypocotyls elongation of *Arabidopsis* seedlings. We found all the test compounds exhibited high inhibitory activity on *Arabidopsis* seedlings elongation. The hypocotyls length *Arabidopsis* seedlings treated with **7a** to **7d** and **7g** are approximately 45.9 ± 2.5, 37.6 ± 3.2, 32.5 ± 3.2, 56.7 ± 1.1 and 21.2 ± 2.3% of non-chemically treated seedlings, respectively, while the positive control of **YCZ-14** was approximately 18.2 ± 2.0%, and that of **Brz** was 56.0 ± 3.3%, respectively. This result indicate that at a concentration of 0.5 μM of chemicals, **YCZ-14** and **7g** strongly inhibited hypocotyls elongation of *Arabidopsis* seedlings, while **Brz **inhibited hypocotyls elongation at a degree about 56%. This result clearly indicates that the potency of **YCZ-14** and **7g** are greater than that of Brz on inhibition hypocotyls elongation in *Arabidopsis* seedlings. Co-application of BL (10 nM) showed different recovery to different test compounds. Among the compounds listed in Table 2, all the **YCZ** analogues showed good recovery to BL, the hypocotyls length were 99.8 ± 4.4, 95.4 ± 4.9, 101.2 ± 4.5, 103.3 ± 4.5 and 104.1 ± 3.3% of none chemical treated control, respectively, while the recovery degree of **Brz** is 63.0 ± 6.1%. Co-application of GA (1 μM) did not show significant recovery for **YCZ** analogues (from 31 to 51%), while the recovery of **Brz** for **GA** treatment is 61.8 ± 2.3%. Interestingly, **YCZ-14** not only inhibited hypocotyls elongation of *Arabidopsis* seedlings, it also induced morphological changes of *Arabidopsis* seedlings grown in the dark (As shown in Figure 2). These physiological changes can be counteracted by application of BL (Figure 2C) but not GA (Figure 2B).

## 3. Experimental

### 3.1. General

Chemicals for synthesis were purchased from Kanto Chemicals Co. Ltd. (Tokyo, Japan) and Tokyo Kasei Co. Ltd. (Tokyo, Japan). Reagents are of the highest grade commercially available. Melting points (mp) were determined with a Yanako melting point apparatus (Tokyo, Japan). ^1^H-NMR spectra were recorded with a JEOL ECP-400 spectrometer (Tokyo, Japan), chemical shifts being expressed in ppm downfield from TMS as an internal standard. High resolution electrospray ionization Fourier transform ion cyclotron resonance mass spectra (ESI-FTICR) were recorded on an Exactive MS System (Thermo Fisher Scientific, Waltham, MA, USA).

### 3.2. Synthesis

*Preparation of 1-phenyl-2-(1,2,4-triazol-1-yl)ethanone * (**2a**). Compound **2a** was prepared using phenacyl bromide as the starting material, as described previously [23]. To a vigorously stirred suspension of 1*H*-1,2,4-triazole (5.52 g, 0.08 mol) and phenacyl bromide **1a** (9.95 g, 0.05 mol) in 30 mL acetone, was added triethylamine (8.1 g, 0.05 mol) dropwise over a period of 1 h with the temperature kept below 0 °C, and the reaction mixture was stirred for another 30 min at room temperature. The mixture was filtered to remove triethylamine hydrobromide salt precipitates, the precipitates was washed with 3 × 10 mL acetone, the combined washings and filtrate were evaporated under reduced pressure, and the residues were dissolved in 50 mL chloroform and then washed with 2 × 25 mL water. After evaporation of chloroform, the yellow solid was recrystallized with 2-propanol, and 1-phenyl-2-(1,2,4-triazol-1-yl) ethanone (**2a**) was obtained as a white solid with a yield of 22.7%. ^1^H-NMR (CDCl_3_), δ: 5.69 (s, 2H), 7.55 (t, *J* = 7.8 Hz, 2H), 7.68 (t, *J *= 7.5 Hz, 1H), 8.00 (d, *J* = 7.8 Hz, 2H), 8.03 (s, 1H), 8.26 (s, 1H). Other compounds **2b**–**h** were prepared in a similar way by the reaction of triazole with the corresponding phenacyl bromide.

*1-(4-Methylphenyl)-2-(1,2,4-triazol-1-yl)ethanone *(**2b**). Yield: 61.0%. ^1^H-NMR (CDCl_3_), δ: 2.46 (s, 3H), 5.66 (s, 2H), 7.34 (d, *J* = 8.1 Hz, 2H), 7.89 (d, *J* = 8.1 Hz, 2H), 8.02 (s, 1H), 8.25 (s, 1H).

*1-(4-Fluorophenyl)-2-(1,2,4-triazol-1-yl)ethanone *(**2c**).Yield: 50.6%. ^1^H-NMR (CDCl_3_), δ: 5.66 (s, 2H), 7.21~7.25 (m, 2H), 8.02 (s, 1H), 8.03~8.07 (m, 2H), 8.25 (s, 1H).

*1-(4-Trifluoromethylphenyl)-2-(1,2,4-triazol-1-yl)ethanone *(**2d**). Yield: 19.8%. ^1^H-NMR (CDCl_3_), δ: 5.71 (s, 1H), 7.83 (d, *J* = 8.1 Hz, 2H), 8.04 (s, 1H), 8.12 (d, *J* = 8.8 Hz, 2H), 8.26 (s, 1H).

*1-Biphenyl-4-yl-2-(1,2,4-triazol-1-yl)ethanone* (**2e**). Yield: 48.7%. ^1^H-NMR (CDCl_3_), δ: 5.67 (s, 2H), 7.37~7.59 (m, 3H), 7.70 (d, *J* = 8.1 Hz, 2H), 7.70 (d, *J* = 8.1 Hz, 2H), 8.01 (d, *J* = 4.0 Hz, 2H), 8.02 (s, 1H), 8.32 (s, 1H).

*1-Naphthalen-2-yl-2-(1,2,4-triazol-1-yl)ethanone* (**2f**). Yield: 52.1%. ^1^H-NMR (CDCl_3_), δ: 5.84 (s, 2H), 7.60~7.70 (m, 2H), 7.91~8.03 (m, 5H), 8.32 (s, 1H), 8.52 (s, 1H).

*1-(2,4-Dichlorophenyl)-2-(1,2,4-triazol-1-yl)ethanone *(**2g**). Yield: 52.6%. ^1^H-NMR (CDCl_3_), δ: 5.33 (s, 2H), 6.92 (s, 1H), 7.02 (s, 1H), 7.38 (d, *J* = 9.1 Hz, 1H), 7.51 (s, 2H), 7.57 (d, *J* = 9.1 Hz, 1H). 

*1-(3,4-Dichlorophenyl)-2-(1,2,4-triazol-1-yl)ethanone *(**2h**). Yield: 65.2%. ^1^H-NMR (CDCl_3_), δ: 5.65 (s, 2H), 7.65 (d, *J* = 8.4 Hz, 1H), 7.83 (dd, *J*_1_ = 2.2 Hz, *J*_2_ = 8.4 Hz, 1H), 8.03~8.09 (m, 1H), 8.21 (s, 1H), 8.25 (s, 1H).

*Preparation of 2,2-dimethyl-4-(4-methylbenzenesulfonate)-1,3-dioxolane-4-methanol *(**4**). A dry 50 mL round-bottomed flask was charged with *p*-toluenesulfonyl chloride (7.1 g, 37 mmol), to which pyridine (15 mL) was added while stirring under nitrogen with a magnetic stir bar. The reaction flask was placed inside a container; an ice/water mixture could be added in the event that the reaction becomes too exothermic. 2,2-Dimethyl-1,3-dioxolane-4-methanol (3.29 g, 25 mmol) was added slowly and stirred for 1.5 h. The mixture was slowly added to a vigorously stirred mixture of de-ionized water (70 mL) and crushed ice (70 g) in a 300 mL Erlenmeyer flask and allowed to stir for an additional 0.5 h. The white precipitate was collected on filter paper and washed with cold water (H_2_O). The product was dried under high vacuum and/or anhydrous sodium sulfate to obtain target compound **4** (4.15 g, 58.0% yield). ^1^H-NMR (CDCl_3_), δ: 1.32 (s, 3H), 1.34 (s, 3H), 2.46 (s, 3H), 3.77 (dd, J = 4.9, 9.0 Hz, 1H), 3.95–4.06 (m, 3H), 4.25–4.31 (m, 1H), 7.35 (d, J = 8.1 Hz, 2H), 7.80 (d, J = 8.4 Hz, 2H).

*Preparation of 1-tosyloxy-2, 3-propanediol *(**5**). Compound **4** (8.21 g, 30 mmol) was dissolved in methanol (300 mL). Then, 0.5 *N* hydrochloric acid (30 mL) was added, and the resulting mixture was heated to reflux. Acetone and methanol were slowly distilled off. Additional methanol (50 mL) and 0.5 *N *hydrochloric acid (20 mL) was added, and the mixture was kept at room temperature until ketal hydrolysis was completed. The mixture was diluted with saturated sodium bicarbonate solution and extracted with EtOAc (3 × 80 mL). The EtOAc extracts were combined, washed with brine (20 mL), dried over anhydrous sodium sulfate, filtered, concentrated, and purified by flash chromatography on silica gel (EtOAc/hexanes = 7:3), affording target compound 5 (4.57 g, 64.8% yield). ^1^H-NMR (CDCl_3_), δ: 2.46 (s, 3H), 3.63 (dd, *J *= 5.1, 11.4 Hz, 1H), 3.71 (dd, *J *= 4.2, 11.5 Hz, 1H), 3.92–3.99 (m, 1H), 4.06–4.13 (m, 2H), 7.37 (d, *J *= 6.6 Hz, 2H), and 7.81 (d, *J *= 6.6 Hz, 2H).

*Preparation of 2RS,4RS-toluene-4-sulfonic acid 2-phenyl-2-1,2,4-triazol-1-ylmethyl-1,3-dioxolan-4-ylmethyl ester* (**6a**). Trifluoromethanesulfonic acid (1.5 mL, 16 mmol) was added to a solution of 1-tosyloxy-2, 3-propanediol (**5**, 1 g, 4.0 mmol) and ketone **2a** (1 g, 3.9 mmol) in toluene (10 mL) under nitrogen. Then, the reaction mixture was stirred at room temperature for 60 h. The reaction was quenched by adding saturated sodium bicarbonate solution (25 mL), extracted with EtOAc (3 × 30 mL), washed with brine, and dried over anhydrous sodium sulfate. The solvent was removed, and the residue was re-dissolved in 2 mL EtOAc. *p*-Toluensulfonic acid monohydrate (750 mg, 3.9 mmol) in EtOAc (2 mL) was added dropwise to preferentially precipitate **6** as a white solid. The mixture was stirred for 30 min and filtered to obtain target compound salt **6**, which was recrystallized from acetonitrile (49.5% yield). ^1^H-NMR (CD_3_OD), δ: 2.36 (s, 3H), 2.46 (s, 3H), 3.62 (dd, *J *= 5.1, 8.4 Hz, 1H), 3.73–3.80 (m, 2H), 3.94–3.97 (m, 1H), 4.20–4.22 (m, 1H), 4.77 (s, 2H), 7.22–7.24 (m, 2H), 7.41–7.51 (m, 6H), 7.69–7.71 (m, 2H), 7.76–7.78 (m, 2H), 8.50 (s, 1H), and 9.29 (s, 1H).

Other compounds **6b**–**h** were prepared in a similar way by the reaction of **5** with the corresponding ketone **2**.

*2RS,4RS-Toluene-4-sulfonic acid 2-(4methylphenyl)-2-1,2,4-triazol-1-ylmethyl-1,3-dioxolan-4-yl-methyl ester *(**6b**). (23.1%). ^1^H-NMR (CD_3_OD), δ: 2.35 (d, *J* = 7.3 Hz, 6H), 3.60 (dd, *J *= 4.9, 8.6 Hz, 1H), 3.71–3.79 (m, 2H), 3.92–3.95 (m, 1H), 4.17–4.20 (m, 1H), 4.73 (s, 2H), 7.41 (d, *J* = 8.1 Hz, 2H), 7.46 (d, *J* = 8.4 Hz, 2H), 7.70 (d, *J* = 8.4 Hz, 2H), 7.76 (d, *J *= 8.1 Hz, 2H), 8.66 (s, 1H), 9.53 (d, 1H).

*2RS,4RS-Toluene-4-sulfonic acid 2-(4-fluorophenyl)-2-1,2,4-triazol-1-ylmethyl-1,3-dioxolan-4-yl-methyl ester *(**6c**). (36.5%). ^1^H-NMR (CDCl_3_), δ: 2.47 (s, 3H), 3.49–3.53 (m, 1H), 3.69 (dd, *J* = 4.4, 8.8 Hz, 1H), 3.77–3.83 (m, 2H), 4.21–4.27 (m, 1H), 4.45 (s, 2H), 7.02–7.08 (m, 2H), 7.37–7.41 (m, 4H), 7.77–7.81 (m, 3H), 8.10 (s, 1H).

*2RS,4RS-Toluene-4-sulfonic acid 2-(4-trifluoromethylphenyl)-2-1,2,4-triazol-1-yl-methyl-1,3-di-oxolan-4-yl-methyl ester *(**6d**). (36.1%). ^1^H-NMR (CDCl_3_), δ: 2.48 (s, 3H), 3.55 (dd, *J* = 5.6, 10.6 Hz, 1H), 3.78–3.85 (m, 3H), 4.22–4.28 (m, 1H), 4.48 (s, 2H), 7.36–7.41 (m, 2H), 7.54–7.69 (m, 4H), 7.78–7.82 (m, 3H), 8.11 (s, 1H).

*2RS,4RS-Toluene-4-sulfonic acid 2-(biphenyl-4-yl)-2-1,2,4-triazol-1-yl-methyl-1,3-dioxolan-4-yl-methyl ester *(**6e**). (42.7%). ^1^H-NMR (CDCl_3_), δ: 2.48 (s, 3H), 3.48 (dd, *J* = 6.2, 10.3 Hz, 1H), 3.69 (dd, *J* = 4.4, 8.8 Hz, 1H), 3.79–3.86 (m, 2H), 4.27–4.30 (m, 1H), 4.50 (d, *J* = 1.5 Hz, 2H), 7.37–7.41 (m, 3H), 7.43–7.50 (m, 4H), 7.57–7.60 (m, 4H), 7.79 (d, *J *= 8.4 Hz, 2H), 7.83 (s, 1H), 8.10 (s, 1H).

*2RS,4RS-Toluene-4-sulfonic acid 2-(naphthalen-2-yl)-2-1,2,4-triazol-1-yl-methyl-1,3-dioxolan-4-yl-methyl ester *(**6f**). (26.3%). ^1^H-NMR (CDCl_3_), δ: 2.48 (s, 3H), 3.53 (dd, *J* = 6.2, 10.6 Hz, 1H), 3.61–3.65 (m, 1H), 3.81–3.86 (m, 2H), 4.28–4.30 (m, 1H), 4.56 (d, *J* = 2.2 Hz, 2H), 7.39 (dd, *J* = 8.1, 11.7 Hz, 3H), 7.49–7.56 (m, 2H), 7.79–7.95 (m, 7H), 8.13 (s, 1H).

*2RS,4RS-Toluene-4-sulfonic acid 2-(2,4-dichlorophyenyl)-2-1,2,4-triazol-1-yl-methyl-1,3-dioxolan-4-yl-methyl ester *(**6g**) (14.0%). ^1^H-NMR (CDCl_3_), δ: 2.47 (s, 3H), 3.51 (dd, *J* = 6.4, 10.4 Hz, 1H), 3.71 (dd, *J* = 4.3, 9.3 Hz, 1H), 3.80–3.85 (m, 2H), 4.25–4.28 (m, 1H), 4.67–4.79 (m, 2H), 7.38–7.47 (m, 4H), 7.77–7.81 (m, 4H), 8.11 (s, 1H).

*2RS,4RS-Toluene-4-sulfonic acid 2-(3,4-dichlorophyenyl)-2-1,2,4-triazol-1-yl-methyl-1,3-dioxolan-4-yl-methyl ester *(**6h**). (45.8%). ^1^H-NMR (CD_3_OD), δ: 2.46 (s, 3H), 3.64 (dd, *J *= 5.1, 8.8 Hz, 1H), 3.76–3.81 (m, 2H), 3.95–3.98 (m, 1H), 4.22–4.24 (m, 1H), 4.79 (s, 2H), 7.23 (d, *J* = 8.1 Hz, 2H), 7.44–7.48 (m, 3H), 7.77 (d, *J *= 8.4 Hz, 2H), 8.62 (s, 1H), 9.48 (s, 1H).

*Preparation of 1-[2-Phenyl-4-(2-trifluoromethoxyphenoxymethyl)-1,3-dioxolan-2-yl-methyl]-1H-1,2,4-triazole (**7a**)*. Potassium hydroxide (160 mg, 2.8 mmol) was added to a solution of tosylate **6** (485 mg, 0.78 mmol) and 2-trifluoromethoxyphenol (133 mg, 0.72 mmol) in dry DMF (5 mL), and the reaction mixture was heated at 50 °C overnight. After cooling to room temperature, the reaction mixture was diluted with water (20 mL) and EtOAc (20 mL), and the organic phase was separated. The aqueous phase was extracted with EtOAc (3 × 20 mL). All of the organic layers were combined, washed with brine (20 mL), dried over anhydrous sodium sulfate, filtered, concentrated, and purified by flash chromatography on silica gel (EtOAc/hexanes = 1:1), affording target compound **7a** (58.9% yield), mp: 100–103 °C. ^1^H-NMR (CD_3_OD), δ: 3.34–3.38 (m, 1H), 3.86–3.95 (m, 3H), 4.40–4.44 (m, 1H), 4.51–4.60 (m, 2H), 6.87–6.89 (m, 1H), 6.95–7.00 (m, 1H), 7.21–7.28 (m, 2H), 7.40–7.44 (m, 3H), 7.51–7.54 (m, 2H), 7.93 (s, 1H), 8.17 (s, 1H). HRMS-ESI calculated for C_19_H_19_N_3_O_3_Na [M+Na]^+^ was 394.0928, and 394.0891 was the experimental value.

Other compounds **7b**–**h** were prepared in a similar method, by reacting 2-trifluoromethoxyphenol with the corresponding **6**.

*1-[2-p-Tolyl-4-(2-trifluoromethoxyphenoxymethyl)-1,3-dioxolan-2-ylmethyl]-1H-1,2,4-triazole* (**7b**).(69.6%). mp: 79.8–81.3 °C. ^1^H-NMR (CDCl_3_); δ: 2.37(s, 3H), 3.33–3.37 (m, 1H), 3.84–3.94 (m, 3H), 4.37–4.43 (m, 1H), 4,49–4.57 (m, 2H), 6.86–6.89 (m, 1H), 6.95–6.99 (m, 1H), 7.20–7.28 (m, 4H), 7.38–7.41 (m, 2H), 7.93 (s, 1H), 8.16 (s, 1H). HRMS-ESI *m/z* calculated for C_21_H_20_F_3_N_3_O_4_Na [M+Na]^+^ 458.1298, found 458.1248.

*1-[2-(4-Fluorophenyl)-4-(2-trifluoromethoxyphenoxymethyl)-1,3-dioxolan-2-ylmethyl]-1H-1,2,4-triazole* (**7c**). (31.6%). mp: 60.8–62.5 °C. ^1^H-NMR (CDCl_3_), δ: 3.40 (dd, *J* = 7.1, 9.3 Hz, 1H), 3.86–3.94 (m, 3H), 4.39–4.43 (m, 1H), 4.53 (d, *J* = 5.1 Hz, 2H), 6.87–6.89 (m, 1H), 6.96–7.05 (m, 1H), 7.06–7.10 (m, 2H), 7.21–7.28 (m, 2H), 7.46–7.50 (m, 2H), 7.91 (s, 1H), 8.16 (s, 1H). HRMS-ESI *m/z* calculated for C_20_H_17_F_4_N_3_O_4_Na [M+Na]^+^ 462.1047, found 462.0999.

*1-[2-(4-Trifluoromethylphenyl)-4-(2-trifluoromethoxyphenoxymethyl)-1,3-dioxolan-2-ylmethyl]-1H-1,2,4-triazole *(**7d**). (28.2%). mp: 93.1–94.5 °C. ^1^H-NMR (CDCl_3_), δ: 3.43–3.48 (m, 1H), 3.83–3.93 (m, 3H), 4.41–4.45 (m, 1H), 4.51–4.60 (m, 2H), 6.88–6.90 (m, 1H), 6.97–7.02 (m, 1H), 7.22–7.29 (m, 2H), 7.63–7.68 (m, 4H), 7.92 (s, 1H), 8.19 (s, 1H). HRMS-ESI *m/z* calculated for C_21_H_17_F_6_N_3_O_4_Na [M+Na]^+^ 512.1015, found 512.0963.

*1-[2-Biphenyl-4-yl-4-(2-trifluoromethoxyphenoxymethyl)-1,3-dioxolan-2-ylmethyl]-1H-1,2,4-triazole *(**7e**). (30.6%). mp: 116.6–117.8 °C. ^1^H-NMR (CDCl_3_), δ: 3.35–3.40 (m, 1H), 3.87–3.91 (m, 2H), 3.96–4.00 (m, 1H), 4.44–4.48 (m, 1H), 4.55–4.64 (m, 2H), 6.88–6.91 (m, 1H), 6.96–7.00 (m, 1H), 7.21–7.29 (m, 2H), 7.36–7.40 (m, 1H), 7.45–7.48 (m, 2H), 7.58–7.64 (m, 6H), 7.95 (s, 1H), 8.21 (s, 1H). HRMS-ESI *m/z* calculated for C_26_H_22_F_3_N_3_O_4_Na [M+Na]^+^ 520.1454, found 520.1404.

*1-Naphthalen-2-yl-4-(2-trifluoromethoxyphenoxymethyl)-1,3-dioxolan-2-ylmethyl]-1H-1,2,4-triazole *(**7f**). (30.5%). mp: 98.5–99.6 °C. ^1^H-NMR (CDCl_3_), δ: 3.39–3.43 (m, 1H), 3.88–3.99 (m, 3H), 4.47–4.48 (m, 1H), 4.59–4.68 (m, 2H), 6.89–7.02 (m, 2H), 7.21–7.28 (m, 2H), 7.51–7.61 (m, 3H), 7.85–8.02 (m, 5H), 8.21 (s, 1H). HRMS-ESI *m/z* calculated for C_24_H_20_F_3_N_3_O_4_Na [M+Na]^+^ was 494.1298, and 494.1248 was found.

*1-[2-(2,4-Dichlorophenyl)-4-(2-trifluoromethoxyphenoxymethyl)-1,3-dioxolan-2-ylmethyl]-1H-1,2,4-triazole *(**7g**). (43.6%). ^1^H-NMR (CDCl_3_), δ: 3.42–3.46 (m, 1H), 3.82–3.98 (m, 3H), 4.40–4.46 (m, 1H), 4.75–4.88 (m, 2H), 6.88–6.90 (m, 1H), 6.96–7.02 (m, 1H), 7.22–7.29 (m, 3H), 7.47–7.55 (m, 2H), 7.90 (s, 1H), 8.19 (s, 1H). HRMS-ESI *m/z* calculated for C_20_H_16_Cl_2_F_3_N_3_O_4_Na [M+Na]^+^ 512.0362, found 512.0310.

*1-[2-(3,4-Dichlorophenyl)-4-(2-trifluoromethoxyphenoxymethyl)-1,3-dioxolan-2-ylmethyl]-1H-1,2,4-triazole *(**7h**). (42.7%) mp: 81.5–82.3 °C. ^1^H-NMR (CDCl_3_), δ: 3.43–3.47 (m, 1H), 3.86–3.96 (m, 3H), 4.40–4.46 (m, 1H), 4.48–4.57 (m, 2H), 6.87–6.90 (m, 1H), 6.97–7.01 (m, 1H), 7.22–7.33 (m, 3H), 7.46–7.48 (m, 1H), 7.60–7.61 (m, 1H), 7.92 (s, 1H), 8.18 (s, 1H). HRMS-ESI *m/z* calculated for C_20_H_16_Cl_2_F_3_N_3_O_4_Na [M+Na]^+^ 512.0362, found 512.0311.

### 3.3. Bioassay Methods for Evaluation Brassinosteroid Biosynthesis Inhibitor

Seeds of *Arabidopsis* (Columbia ecotype) were purchased from Lehle Seeds (Round Rock, TX, USA). The seeds used for the assay were sterilized in 1% NaOCl for 20 min and washed with sterile distilled water. Seeds were sown on a 1% solidified agar medium containing half Murashige and Skoog salt in Petri dishes with or without chemicals. Plants were grown in 16 h light (240 µE/m^2^s) and 8 h dark conditions at 22 °C in a growth chamber with or without added chemicals. For the dark condition, Petri dishes were wrapped in four layers of aluminum foil. The biological activities of the test compounds were measured 5 days after sowing the seeds. The hypocotyl length of 15–20 *Arabidopsis* seedlings was measured with a ruler, and each experiment was repeated at least two times.

## 4. Conclusions

We have reported the synthesis and structure-activity relationships studies of 2*RS*,4*RS*-1-[2-phenyl-4-[2-(2-trifluromethoxyphenoxy)-ethyl]-1,3-dioxolan-2-yl-methyl]-1*H*-1,2,4-triazole derivatives as potent inhibitors of brassinosteroid biosynthesis. The analogues with different aromatic substituent at position 2 of dioxolane were successfully synthesized by a method we described previously [22]. The biological activity of the test compounds were evaluated by a method using *Arabidopsis* seedlings grown in the dark. We found that the variation of aromatic substituent significantly affect the inhibitory potency. Structure-activity relationships studies indicated that a 4-chlorophenyl analogue is the most potent inhibitor of BR biosynthesis, with an IC_50_ value approximately 0.12 ± 0.04 μM, while a bulky biphenyl group exhibited a great negative effect on promoting the inhibitory potency with an IC_50_ larger than 10 μM.
